# Abaloparatide Exerts Bone Anabolic Effects with Less Stimulation of Bone Resorption-Related Factors: A Comparison with Teriparatide

**DOI:** 10.1007/s00223-018-0422-4

**Published:** 2018-05-03

**Authors:** Akito Makino, Hideko Takagi, Yoshimasa Takahashi, Naoki Hase, Hiroyuki Sugiyama, Kei Yamana, Tsunefumi Kobayashi

**Affiliations:** Teijin Institute for Bio-Medical Research, Teijin Pharma Limited, Hino, Tokyo, Japan

**Keywords:** Abaloparatide, Teriparatide, Osteoporosis treatment, Osteoblast

## Abstract

Abaloparatide (ABL) is a novel synthetic peptide analog of parathyroid hormone-related protein. In previous reports, intermittent ABL administration showed robust bone mineral density (BMD) increase and reduced the incidence of fractures in patients with osteoporosis, while its calcemic effect was reduced, as compared with teriparatide (TPTD), a parathyroid hormone N-terminal fragment. The present study aimed to elucidate the effects of ABL on bone anabolism and bone turnover as compared with TPTD. In ovariectomized (OVX) rats, ABL increased the bone strength and BMD of lumbar spine by intermittent administration similar to TPTD. Both ABL and TPTD increased the bone formation marker serum P1NP with little effect on the bone resorption maker urine DPD/Cr, suggesting anabolic effects on bone. In human osteoblastic cells, both peptides increased the expression of bone resorption-related factors such as RANKL/OPG and M-CSF, and the effects of ABL were significantly attenuated as compared with those of TPTD under transient 6-h treatment, although no significant differences were found under continuous treatment. In contrast, ABL and TPTD similarly promoted the expression of bone formation-related factors, IGF-1 and osteocalcin. In addition, there were no significant differences in the effects on WNT signaling inhibitors such as sclerostin and dickkopf-related protein 1 (DKK1) between the two peptides. These results demonstrate that ABL exerts bone anabolic effects in OVX rats. It is also indicated that ABL stimulates the expression of RANKL/OPG and M-CSF less than TPTD, while showing similar effects on bone formation-related factors and WNT signaling inhibitors in vitro. The profile of ABL indicates that it would be a suitable bone anabolic agent for osteoporosis.

## Introduction

Parathyroid hormone (PTH) and PTH-related protein (PTHrP) have a common G protein-coupled receptor, PTH1R, which plays a pivotal role in bone turnover and calcium homeostasis [1]. Numerous studies have revealed that PTH and PTHrP increased bone mass due to the greater acceleration of bone formation than resorption when administrated intermittently [2, 3]. In contrast, continuous infusion caused bone loss and hypercalcemia because bone resorption was predominantly promoted over formation [4, 5]. These findings suggest that PTH and PTHrP are useful for the treatment of certain kinds of bone disease such as osteoporosis when they are administrated appropriately.

In fact, teriparatide (TPTD), the N-terminal 34 amino acids fragment of PTH, is currently the only bone anabolic agent used for the treatment of osteoporosis, by daily or weekly subcutaneous administration [6, 7]. The effect of TPTD on bone mineral density (BMD) is greater than antiresorptive agents such as bisphosphonates. However, its effect on the hip is modest and the usage is limited in patients with the risk of hypercalcemia because blood calcium is mildly increased by the stimulation of bone resorption.

The mechanism of action of TPTD on bone turnover is not fully understood; however, many studies have examined the molecular mechanisms in recent decades. According to a previous study, PTH(1–34) acts on osteoblasts and indirectly induces bone resorption by activating osteoclasts [8]. PTH(1–34) increases the expression of receptor activator of nuclear factor kappa-B ligand (RANKL) and macrophage colony-stimulating factor (M-CSF) and decreases the expression of osteoprotegerin (OPG), a decoy receptor of RANKL [9]. These effects of PTH(1–34) further promote the maturation of osteoclasts with bone resorbing activity. On the other hand, PTH(1–34) promotes the differentiation and mineralization of osteoblasts. This anabolic action of PTH(1–34) is thought to be mediated in part by the enhancement of insulin-like growth factor-1 (IGF-1) expression [10]. Moreover, it has recently been reported that PTH(1–34) also affects osteocytes, the terminal differentiation state of osteoblasts, and that it exerts anabolic action by inhibiting the secretion of WNT signaling antagonists such as sclerostin and dickkopf-related protein 1 (DKK1) [11, 12]. These effects of PTH(1–34) are induced mainly by intracellular cAMP, which is generated after the ligand binds to PTH1R [13].

Abaloparatide (ABL), a novel synthetic peptide analog of PTHrP, has recently approved by Food and Drug Administration as a drug for severe osteoporosis. In the phase 3 ACTIVE clinical trial, daily subcutaneous administration of ABL showed robust BMD increases at the hip as well as the spine, and resulted in a reduced incidence of vertebral and non-vertebral fractures, whereas its calcemic effect was reduced as compared with TPTD [14]. Analysis of bone turnover markers also suggested that ABL increased both bone formation and resorption, although its effects on both makers of bone formation and resorption were attenuated as compared with TPTD [14]. These results suggest that ABL is an anabolic agent in which bone formation is predominantly enhanced. However, why the effect on bone turnover is different between these peptides remains unclear. Although there were some reports on bone anabolic effects of ABL in non-clinical studies [15–17], none of them have evaluated in head-to-head comparison with TPTD.

In the present study, we evaluated the bone anabolic effects of ABL and TPTD by intermittent administration to ovariectomized (OVX) rats. Furthermore, to elucidate the difference between ABL and TPTD on bone turnover, the expression of bone formation and resorption-related factors were evaluated in osteoblastic cells by transient or intermittent treatment.

## Materials and Methods

### Peptides and Cells

Abaloparatide ([Glu^22,25^, Leu^23,28,31^, Aib^29^, Lys^26,30^] human PTHrP(1–34)–NH_2_) was synthesized by IPSEN (Paris, France). Teriparatide (human PTH(1–34)) was purchased from BACHEM (Bubendorf, Switzerland).

### Cell Culture

The human osteoblastic cell line SaOS-2 and the rat osteoblastic cell line UMR-106 were purchased from the European Collection of Cell Cultures (Wiltshire, UK). SaOS-2 and UMR-106 were maintained in growth medium (GM): McCoy’s 5A (for SaOS-2) or Dulbecco’s Modified Eagle’s Medium (for UMR-106) supplemented with 10% FBS and 1% penicillin/streptomycin at 37 °C with 5% CO_2_. To initiate cell differentiation, cells were grown in differentiation medium (DM): GM containing 50 µg/mL ascorbic acid, 10 mmol/L β-glycerophosphate, and 10 nmol/L dexamethasone. The culture medium was exchanged every 6 days or less.

### cAMP Accumulation Assay

Cells were cultured in 96-well plates at a density of 4 × 10^4^ cells/well in GM overnight. The medium was replaced with serum-free GM (containing 0.1% BSA instead of 10% FBS) for culture under starvation conditions. Twenty-four hours after the start of starvation, ABL, TPTD, or vehicle (water containing 0.1% BSA) were added to the cells with 2 mmol/L of 3-isobutyl-1-methylxanthine for 10 min. Cells were lysed and accumulated cAMP was measured using the cAMP-Screen System (Applied Biosystems, Waltham, MA, USA) according to the manufacturer’s instructions.

### Animal Experiment

Ten-week-old female Sprague–Dawley rats were purchased from Charles River Laboratories Japan, Inc. (Kanagawa, Japan) and ovariectomized at 12 weeks old. Thirty-five days after surgery, rats received once daily subcutaneous injection of ABL, TPTD, or vehicle (saline containing 0.1% heat-inactivated rat serum) for 28 days. Day 0 indicates the day when administration was started. On day 26, rats were anesthetized with intraperitoneally administered pentobarbital sodium and the lumbar spine (L4 and L5) BMD was measured with a PIXImus2 (GE Healthcare, Chicago, IL). Blood (collected from the tail vein) and urine samples were obtained on day 28. Prior to collection, rats were fasted for 15–17 h. Rats were euthanized after the blood and urine collection, and the lumbar spine was removed for the measurement of bone strength. All experimental procedures were approved by the Animal Care and Use Committee of Teijin Institute for Bio-Medical Research.

### Measurement of Bone Turnover Markers

Serum P1NP concentration was measured with a Rat/Mouse PINP EIA kit (Immunodiagnostic Systems, Boldon, UK). Urine DPD concentration was measured with an Osteolinks DPD kit (Quidel, San Diego, CA, USA). Urine creatinine (Cr) concentration was measured with a 7180 Autoanalyzer (Hitachi High-Technologies Corporation, Tokyo, Japan).

### Measurement of Bone Strength

L4 was isolated from the lumbar spine. The height of the vertebral body was measured with a vernier caliper. To obtain vertebral body specimens, the cranial and caudal ends of the vertebral body were cut with a diamond saw to a height of 4 mm. Bone strength was measured with a MZ-500S (MARUTO Testing Machine Company, Tokyo, Japan).

### Evaluation of Bone Resorption-Related Factors

SaOS-2 was cultured in 48-well plates at a density of 5 × 10^4^ cells/well in DM for 10 days. The medium was replaced with serum-free DM for culture under starvation conditions. Twenty-four hours after the start of starvation, cells were treated with ABL and TPTD at a concentration of 100 nmol/L. Six hours after treatment, cells were washed with PBS twice, and cultured with peptide-free or peptide-containing medium for the rest of the experimental period.

### Evaluation of Bone Formation-Related Factors

SaOS-2 was cultured in 12-well plates at a density of 2 × 10^5^ cells/well in DM. Concomitant with the initiation of cell differentiation, cells were treated with 100 nmol/L of ABL and TPTD once daily for 11 days. The peptide exposure was limited to the first 6 h of the 24-h incubation cycle, and the medium was replaced with peptide-free medium for the rest of the cycle after washing with PBS twice.

### Evaluation of WNT Signaling Antagonists

SaOS-2 was cultured in 48-well plates at a density of 5 × 10^4^ cells/well in DM for 30 days. Cells were treated with ABL and TPTD at a concentration of 100 nmol/L. Six hours after treatment, cells were washed with PBS twice, and cultured with peptide-free or peptide-containing medium for the rest of the experimental period.

### Quantitative RT-PCR

Cells were lysed and total RNA was extracted using an RNeasy Mini Kit (Qiagen, Valencia, CA, USA). RNA was reverse-transcribed into cDNA using the SuperScript III First-Strand Synthesis System for RT-PCR (Invitrogen, Carlsbad, CA, USA). Quantitative RT-PCR was performed on a 7500 Real-Time PCR System (Applied Biosystems) using Power SYBR Green PCR Master Mix (Thermo Fisher Scientific, Waltham, MA, USA). The primers used in this study were as follows: 5′-CGATGGTGGATGGCTCATG-3′ (forward) and 5′-ACCAGATGGGATGTCGGTG-3′ (reverse) for RANKL, 5′-GATGTGGTGACCAAGCCTGA-3′ (forward) and 5′-CTCAGAGTCCTCCCAGGTCA-3′ (reverse) for M-CSF, 5′-GCCTGGCACCAAAGTAAACG-3′ (forward) and 5′-GCTCGAAGGTGAGGTTAGCA-3′ (reverse) for OPG, 5′-TTTCAAGCCACCCATTGACC-3′ (forward) and 5′-GCGGGTACAAGATAAATATCCAAAC-3′ (reverse) for IGF1, 5′-CACCGAGACACCATGAGAGC-3′ (forward) and 5′-CTGCTTGGACACAAAGGCTGC-3′ (reverse) for osteocalcin, 5′-CCTGTGCTCTCCCAGTAACC-3′ (forward) and 5′-CTTCATTTGCCAAGGGTGGTG-3′ (reverse) for DMP1, 5′-TGGCAGGCGTTCAAGAATGA-3′ (forward) and 5′-TGTACTCGGACACGTCTTTGG-3′ (reverse) for SOST, 5′-TGACAACTACCAGCCGTACC-3′ (forward) and 5′-CAGGCGAGACAGATTTGCAC-3′ (reverse) for DKK1, 5′-GTGAAGGTCGGAGTCAACG-3′ (forward) and 5′-TGAGGTCAATGAAGGGGTC-3′ (reverse) for GAPDH.

### Measurement of M-CSF, Sclerostin and DKK1

Human Quantikine M-CSF, Sclerostin, and DKK1 ELISA kits (R&D Systems, Minneapolis, MN, USA) were used to quantify M-CSF, sclerostin, and DKK1, respectively, in the cell supernatants according to the manufacturer’s instructions.

### Data Analysis

All data are expressed as means ± s.e.m. For calculation of EC_50_ and *E*_max_, 4-parameter logistic curve fitting was performed. Student’s *t* test was used for two-group comparisons. Dunnett’s or Tukey’s test was used for multiple comparisons. Significance was inferred from *P* values of < 0.05. All data were analyzed using GraphPad Prism version 6.03 (GraphPad Software, La Jolla, CA, USA).

## Results

### cAMP Accumulation and Bone Anabolic Effect of ABL

**Fig. 1:**
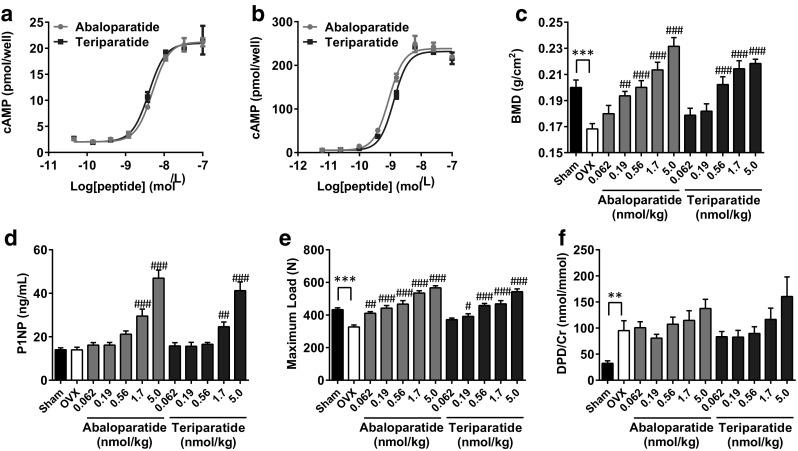
Effect of ABL on cAMP accumulation and bone anabolism. **a, b** SaOS-2 and UMR-106 were incubated with either ABL or TPTD for 10 min. Cells were lysed and accumulated cAMP was measured. *n* = 3 replicate wells per concentration. Data are representative of three independent experiments. **c** OVX rats received once daily subcutaneous injection of ABL or TPTD for 28 days. On day 26, L4–L5 BMD was measured. **d** Results of L4 bone strength, **e** serum P1NP concentration, and **f** urine DPD/Cr concentration on day 28. **P < 0.01, ***P < 0.001, versus Sham. ^#^*P* < 0.05, ^##^*P* < 0.01, ^###^*P* < 0.001, versus OVX. For **b**–**e**, *n* = 8 or 9 per group. Data are representative of two independent experiments

We first evaluated the effect of ABL on cAMP accumulation in human and rat osteoblastic cells. SaOS-2 and UMR-106 were treated with these peptides for 10 min and accumulated cAMP was measured. As expected, ABL as well as TPTD dose-dependently increased cAMP in both human (Fig. 1a) and rat osteoblastic cells (Fig. 1b). There were no significant differences in EC_50_ and *E*_max_ values between the two peptides in SaOS-2, whereas the EC_50_ value of ABL was slightly (~ 1.5-fold) greater than that of TPTD in UMR-106 (Table 1).

**Table 1:** Effect of ABL and TPTD on cAMP accumulation

	Abaloparatide	Teriparatide	*P* value
SaOS-2			
EC_50_	5.1 ± 0.4	3.9 ± 0.5	0.13
*E*_max_	21.9 ± 1.4	22.0 ± 1.5	0.96
UMR-106			
EC_50_	0.84 ± 0.03	1.18 ± 0.06	0.0067
*E*_max_	278.4 ± 20.8	271.4 ± 25.0	0.84

EC_50_ and *E*_max_ were expressed as nmol/L. Data were shown from three independent experiments, each performed triplicate

Next, to assess the bone anabolic effect of ABL, the peptide was administrated intermittently to OVX rats. Four weeks of subcutaneous injection of ABL and TPTD significantly increased bone strength as well as BMD of the lumbar spine, consistent with cAMP accumulation (Fig. 1c, d). The bone formation marker serum P1NP was significantly increased following either ABL or TPTD administration, suggesting that the two peptides had anabolic effects on bone (Fig. 1e). In contrast, there were no significant differences in the urinary bone resorption marker DPD/Cr following administration of the two peptides, although a slight increase was observed at 5.0 nmol/kg (Fig. 1f).

### Effects on Bone Resorption-Related Factors in Osteoblastic Cells

**Fig. 2:**
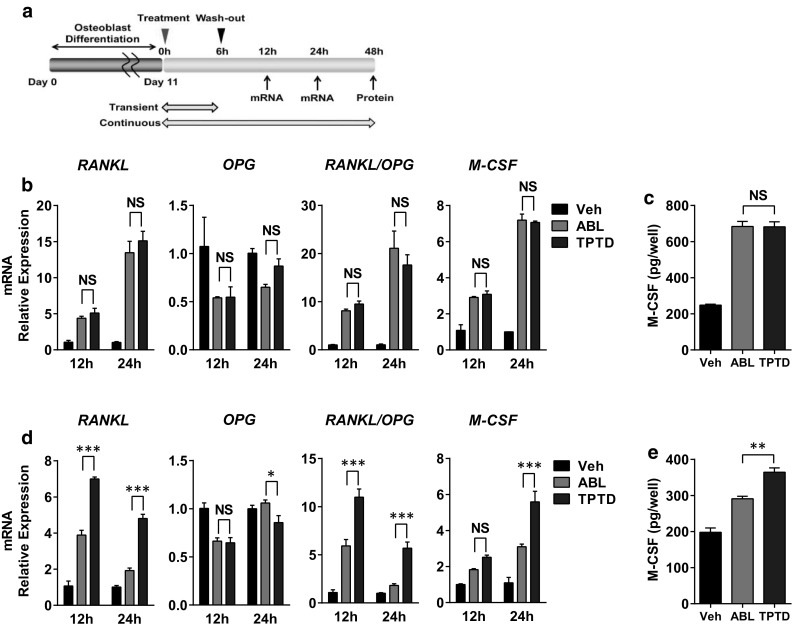
Effects of ABL and TPTD on bone resorption-related factors in osteoblastic cells. **a** Schema of the experiment. SaOS-2 was incubated with either ABL or TPTD at a concentration of 100 nmol/L on day 10. Six hours after treatment, the medium was replaced with peptide-free or peptide-containing medium for the rest of the experimental period. **b, c** Analysis of relative gene expression and protein secretion by qRT-PCR and ELISA under continuous treatment. **d, e** Analysis of relative gene expression and protein secretion by qRT-PCR and ELISA under transient treatment. *P < 0.05, **P < 0.01, ***P < 0.001. *N.S*. not significant. *n* = 3 replicate wells per group. Data are representative of two independent experiments

Due to the poor efficacy of the two peptides on bone resorption maker in our rat study, we evaluated the effects of the two peptides on bone turnover in vitro. As mentioned above, the effects of the peptides on bone turnover drastically change depending on the exposure time. Thus, SaOS-2 differentiated into mature osteoblastic cells were treated with ABL and TPTD under a continuous or transient condition, and the effects on bone resorption-related factors were compared (Fig. 2a). As a result, ABL and TPTD similarly promoted RANKL, RANKL/OPG, and M-CSF, but inhibited OPG mRNA expression in the cells treated throughout the experimental period (Fig. 2b). The protein secretion of M-CSF in the medium was also increased following treatment with the two peptides, and their effects did not differ significantly (Fig. 2c). On the other hand, when the treatment was limited to the first 6 h and the unbound ligands were washed out thereafter, the effects of ABL on bone resorption-related factors were significantly attenuated as compared with TPTD (Fig. 2d). These tendencies were also observed for M-CSF protein secretion, as the amount of protein in the medium was significantly less with ABL treatment than with TPTD (Fig. 2e).

### Effects on Bone Formation-Related Factors in Osteoblastic Cells

**Fig. 3:**
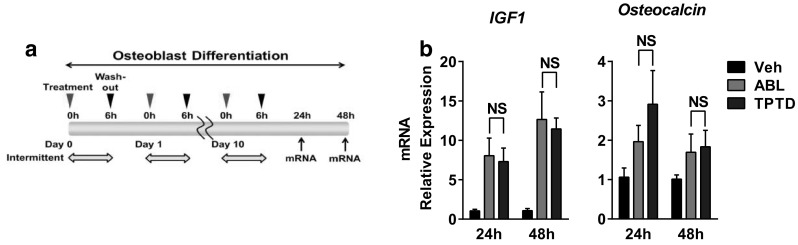
Effects of ABL and TPTD on bone formation-related factors in osteoblastic cells. **a** Schema of the experiment. Cells were treated with either ABL or TPTD at a concentration of 100 nmol/L once daily for 11 days. The treatment was limited to the first 6 h of the 24-h incubation cycle. **b** Analysis of relative gene expression by qRT-PCR. *N.S*. not significant. *n* = 3 replicate wells per group. Data are representative of two independent experiments

We next investigated the effect of ABL on bone formation in SaOS-2. In the previous study, bone formation related to PTH1R signaling was enhanced by the intermittent treatment of TPTD in osteoblasts [18]. This anabolic effect of intermittent TPTD treatment was mediated by IGF-1, which promoted the differentiation and mineralization of osteoblasts [18]. We therefore evaluated the effects on IGF-1 and osteocalcin, a marker gene for differentiated osteoblasts, with a repeating 6-h exposure cycle of ABL and TPTD in SaOS-2 (Fig. 3a). As expected, ABL as well as TPTD increased the gene expression of IGF-1 and osteocalcin (Fig. 3b). The effects of the two peptides were not significantly different, in contrast to the results of bone resorption-related factors under transient treatment. We could hardly detect IGF-1 and osteocalcin expression with continuous treatment of these peptides (data not shown), consistent with a previous study [18].

### Effects on WNT Signaling Antagonists in Osteocyte-Like Cells

**Fig. 4:**
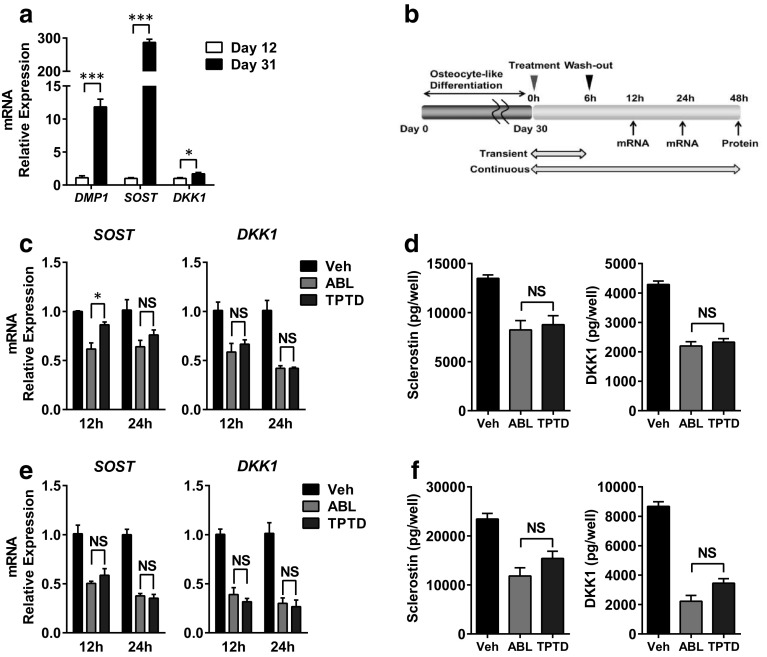
Effects of ABL and TPTD on WNT signaling inhibitors in osteocyte-like cells. **a** SaOS-2 was cultured under differentiation conditions for 9 or 28 days. Seventy-two hours following the last medium replacement, relative gene expression was measured by qRT-PCR. *n* = 3–4 replicate wells per group. Data are representative of two independent experiments. **b** Schema of the experiment regarding (**c**–**f**). SaOS-2 was incubated with either ABL or TPTD at a concentration of 100 nmol/L on day 30. Six hours after treatment, the medium was replaced with peptide-free or peptide-containing medium for the rest of the experimental period. **c, d** Analysis of relative gene expression and protein secretion by qRT-PCR and ELISA under continuous treatment. **e, f** Analysis of relative gene expression and protein secretion by qRT-PCR and ELISA under transient treatment. *P < 0.05, ***P < 0.001, *N.S*. not significant. For **c** to **f**, *n* = 3 replicate wells per group. Data are representative of two independent experiments

Finally, we evaluated the effects of ABL on WNT signaling antagonists. WNT signaling antagonists such as sclerostin and DKK1 are secreted from osteocytes. They inhibit bone formation by antagonizing canonical WNT ligands and subsequently suppress WNT-mediated signaling pathways [19]. Previous studies revealed that osteocytes also express PTH1R and that TPTD inhibited the expression of WNT signaling antagonists in osteocytes [11, 12]. We therefore evaluated the effects of ABL on WNT signaling antagonists in comparison with TPTD. It is generally known that human osteocytes have been difficult to obtain because of the lack of established cell lines till now. Recently, it was reported that SaOS-2 showed osteocyte characteristics following long-term culture under differentiation conditions [20]. In our experimental condition, the elevation of osteocyte marker genes was confirmed when SaOS-2 was cultured for 31 days as compared with cells grown for 12 days (Fig. 4a). By using osteocyte-like SaOS-2, the effects on WNT signaling antagonists were evaluated under transient or continuous treatment conditions (Fig. 4b). As a result, ABL and TPTD similarly suppressed sclerostin and DKK1 gene expression when the treatment was limited to the first 6 h (Fig. 4c). The protein secretion of sclerostin and DKK1 was also inhibited by the two peptides, and their efficacies were not significantly different (Fig. 4d). It should be noted that the efficacy of the two peptides on WNT signaling antagonists was almost the same regardless of the treatment condition, as these peptides similarly inhibited sclerostin and DKK1 even under continuous treatment (Fig. 4e, f).

## Discussion

In the present study, we evaluated the effects of ABL on bone anabolism and bone turnover as compared with TPTD. ABL increased cAMP accumulation in osteoblastic cells, and also increased BMD in OVX rats by intermittent administration similar to TPTD. The effects of ABL on bone were suggested to be anabolic, as evidenced by the increased serum P1NP. These results were consistent with the clinical finding that this peptide had potent efficacy in patients with osteoporosis.

Unlike the clinical study [14], the maker of bone resorption was poorly affected by both peptides in our OVX experiment, which made us difficult to compare the influence on bone turnover. One major possible reason for the difference is due to the use of young rats in our study. In contrast to postmenopausal women with osteoporosis, young rats continue to growth and show the high level of bone remodeling. These profiles of young rats may mask the effect of the two peptides on bone resorption. Another reason may be the short duration of the treatment period or insufficient treatment frequency optimized for rodents. Bone histomorphometrical analysis may be required to detect and compare the bone resorption effect between ABL and TPTD in this study.

We therefore used human osteoblastic cells to evaluate the effects on bone resorption- and formation-related factors in vitro. We found that the effects of ABL on bone resorption-related factors were significantly attenuated when the treatment was limited to the first 6 h as compared with TPTD. These results suggest that ABL stimulates bone resorption less than TPTD under transient treatment; however, the influence of the two peptides on osteoclastogenesis requires elucidation.

It is unclear why ABL stimulates lower expression of bone resorption-related factors than TPTD with transient treatment only. Recently, it was reported that PTH1R has at least two types of active conformations and that the binding conformation selectivity differs between ABL and TPTD [21, 22]. This difference further affects the duration of the downstream signaling. As a result, cAMP production by TPTD is sustained after the unbound ligand is washed out, while that by ABL is transient [22]. We therefore thought that the difference in bone resorption-related factors could arise from the duration of cAMP elevation between the two peptides.

There is another possibility that the two peptides could show the different activity on protein kinase C (PKC). PKC is the downstream signal of PTH1R and is involved in the stimulation of bone resorption-related factors and bone catabolism [23]. In fact, ostabolin, an amidated form of human PTH(1–31), differs from PTH(1–34) in PKC activation [24]. It showed cAMP accumulation without activating PKC, while PTH(1–34) stimulated both cAMP and PKC [24]. The effects of ostabolin on bone turnover look like that of ABL to some extent; ostabolin stimulated bone formation similar to PTH(1–34), with less effect on bone resorption than PTH(1–34) in the preclinical study [25]. Further research would be required on the effect of ABL on PKC activation for fully understanding of mechanism of action.

It should be noted that there were no significant differences in IGF-1 and osteocalcin expression between the two peptides following the 6-h intermittent treatment. Similarly, the effects of the two peptides on sclerostin and DKK1 expression were almost the same regardless of the exposure time. These results suggest that the effects on bone formation-related factors and WNT signaling inhibitors are comparable between ABL and TPTD even with intermittent/transient treatment, unlike those on bone resorption-related factors.

A different regulation system appears to exist between the bone resorption- and formation-related factors. One hypothesis is that these factors are regulated by the balance between the duration and intensity of cAMP signaling. In other words, the bone resorption-related factors were solely dependent on the duration of cAMP generation, while the bone formation-related factors including WNT signaling inhibitors were affected by the amount of cAMP rather than duration. Notably, mice injected with a PTH analog modified to prolong cAMP generation exhibited highly increased bone resorption and blood calcium concentration [26]. Further research will be required to clarify whether downstream signaling was influenced by the shifting balance between cAMP duration and intensity.

The half-life of ABL and TPTD in humans was approximately an hour or less following subcutaneous injection [27, 28], suggesting almost all of the peptides were degraded in the first several hours. It is likely that our results using transient/intermittent 6-h treatment reflect the outcome of the clinical study. In the ph3 ACTIVE study, subcutaneous injection of ABL increased the bone formation marker with less stimulation of the bone resorption marker than observed for TPTD [14].

In conclusion, our study demonstrated that ABL increased BMD as well as bone strength by enhancing bone formation similar to TPTD. It was also suggested that ABL stimulated the expression of RANKL/OPG and M-CSF less than TPTD under transient 6-h treatment, while bone formation-related factors and WNT signaling inhibitors were similarly enhanced. The profile of ABL indicates that it would be a suitable bone anabolic agent for osteoporosis.
